# Arsenic trioxide improves hematopoiesis in refractory severe aplastic anemia

**DOI:** 10.1186/1756-8722-5-61

**Published:** 2012-10-09

**Authors:** Ning Li, Yongping Song, Jian Zhou, Baijun Fang

**Affiliations:** 1 Henan Key Lab of Experimental Haematology, Henan Institute of Haematology, Henan Tumor Hospital affiliated to Zhengzhou University, Zhengzhou, China; 2 Henan Institute of Haematology, Henan Tumor Hospital affiliated to Zhengzhou University, 127 Dongming Road, Zhengzhou, 450008, China

**Keywords:** Aplastic anemia, Hematopoiesis, Arsenic trioxide, Adipocytes, Mesenchymal stem cells

## Abstract

We investigated the efficacy of arsenic trioxide (ATO) in patients with refractory severe aplastic anemia (SAA). A total of 5 consecutive adults were enrolled. The patients received ATO at a dose of 0.15 mg/kg intravenously daily for 5 days every week for 8 weeks. If necessary, a second course was performed after an interval of one week. All patients achieved clinically significant responses to ATO. The overall complete response rate and overall response rate at 17 weeks were 60% (3/5) and 100%(5/5), respectively. So treatment with ATO may be a feasible approach in patients with refractory SAA.

## To the editor

No standard therapies are available for patients who have severe aplastic anemia (SAA) that is refractory to immunosuppressive therapy and are ineligible for hematopoietic stem cell transplantation (HSCT). For such patients, an alternative protocol is urgently needed.

From May 2009 to June 2010, a total of 5 consecutive adults (age range, 21–43 years) with a diagnosis of SAA, defined according to standard criteria [1], entered into this study. All of them failed one or two courses of horse or rabbit anti-thymocyte globulin/cyclosporine-based regimens and all of them did not have a suitable donor for HSCT . Other eligibility criteria included adequate hepatic functions, adequate renal function, and adequate cardiac status. The study was approved by the Institutional Review Board.

None of the patients received any immunosuppressive or cytokine therapy for at least 2 month prior to enrollment. Eligible patients received arsenic trioxide (ATO) at a dose of 0.15 mg/kg intravenously daily for 5 days every week for 8 weeks. If necessary, a second course was performed after an interval of one week. Complete response (CR) was defined as satisfaction of all three peripheral blood count criteria: (1) absolute neutrophil count (ANC) > 1 × 10^9^/L; (2) haemoglobin > 10 g/dL; (3) platelet count > 100 × 10^9^/L. Partial response (PR) was defined as transfusion independence associated with ANC greater than 0.5 × 10^9^/L, haemoglobin greater than 8 g/dL, and platelet count greater than 30 × 10^9^/L. Transfusion dependence was taken as evidence of no response. Relapse was indicated by the requirement for red blood cells or platelets transfusion after having been independent from transfusions for at least 3 months.

**Figure 1:**
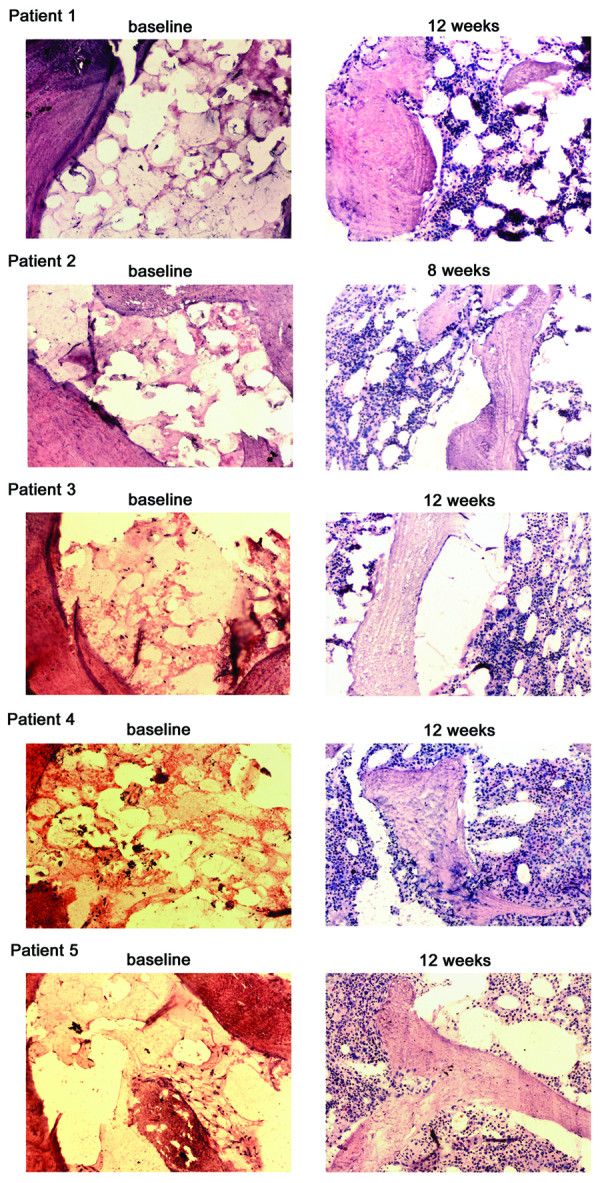
**Hematopoietic recovery in five patients with refractory aplastic anemia after arsenic trioxide therapy.** Bone marrow biopsy specimens were obtained from the five patients. Specimens from pre- and post-treatment (at 8 weeks) were shown. (Hematoxylin and eosin stain; Original magnification: × 100).

The clinical characteristics of patients and outcomes after ATO treatment are summarized in Tables 1 and 2. The overall response rate at 8 weeks was 100% (5/5) after the initiation of treatment, including 20% (1/5) CR and 80% (4/5) PR. The median time to initial response was 43 days (range, 41– 48 days). Four patients with a PR received a second course of ATO and continued to have clinically significant improvements in blood counts. Two of them eventually met response criteria for CR at 17 weeks after the initiation of treatment. So the overall CR rate and overall response rate at 17 weeks were 60% (3/5) and 100%(5/5), respectively. Serial bone marrow biopsies showed hematopoietic recovery accompanied by a decrease in adipocyte number in patients after getting a response (Figure 1). Actuarial survival was 100% at 1 year and 80% at 2 years. No patient showed evidence of clonal evolution or cytogenetic abnormalities at the last follow-up visit.

**Table 1:** Characteristics of patients and outcomes after ATO treatment

**Patient no.**	**Age/gender**	**Time since diagnosis (months)**	Previous Courses of intensive IST^*^(months)	**Time since last intensive IST (months)**	**Response to prior intensive IST**	**Time to initial response to ATO (days)**	**Time to maximum response to ATO (days)**	**The final response to ATO**	**Present status**
1	21/M	35	2	9.8	Primary refractory^#^	41	56	CR	CR and well 34 months
2	37/F	71	1	11	Relapsed refractory^†^	43	102	PR	PR and well 28 months
3	43/M	38	2	13.4	Primary refractory	42	87	CR	CR and well 25 months
4	35/F	41	2	9.2	Primary refractory	46	69	CR	Relapsed and transfusion dependent
5	29/M	63	2	10.3	Primary refractory	48	108	PR	PR and well 20 months

M, male; F, female; IST, Intensive immunosuppressive therapy; ATO, arsenic trioxide; CR, complete response; PR, partial response.

^*^IST regimens included horse antithymocyte globulin (ATG), rabbit ATG, cyclophosphamide, and fludarabine.

^#^Primary refractory disease was defined as no prior adequate response to IST.

^†^Relapsed refractory disease was defined as a prior adequate response to at least one regimen of IST.

**Table 2:** Patients’ characteristics before and after ATO treatment

**Patient No.**	**No. of RBC transfusions per week (mean units)**		**platelet count (×10**^**9**^**/L)**		**Haemoglobin (g/dL)**		**ANC (× 10**^**9**^**/L)**	
	**Before ATO therapy**	**After maximum response to ATO**	**Before ATO therapy**	**After maximum response to ATO**	**Before ATO therapy**	**After maximum responseto ATO**	**Before ATO therapy**	**After maximum response to ATO**
1	1.8	Transfusion independence	9	115	5.1	11.4	0.2	2.0
2	1.9	Transfusion independence	11	73	5.3	8.6	0.1	0.8
3	2.3	Transfusion independence	14	138	4.9	11.2	0.3	1.9
4	2.6	Transfusion independence	9	107	6.1	12.3	0.2	2.2
5	1.9	Transfusion independence	5	67	5.7	8.8	0.2	0.9

RBC, red blood cells; ATO, arsenic trioxide; ANC, absolute neutrophil count.

ATO-related toxicities occurred in 1 of 5 with skin reactions (rash, itching, erythema), 2 of 5 with gastrointestinal reactions (vomiting, nausea, diarrhea), 1 of 5 with liver dysfunction, and 2 of 5 with facial edema. All the side effects were modest and responded to symptomatic treatment. No patient discontinued therapy because of ATO-related toxicities.

Studies suggest that bone marrow adipocytes are predominantly negative regulators of the bone marrow microenvironment [2]. Bone marrow adipocytes are less supportive of hematopoiesis than those of other cell types derived from mesenchymal progenitors such as bone marrow myofibroblasts or osteoblasts [3,4]. In addition, it has been shown that ablation of the bone marrow adipocyte compartment can induce osteogenesis [2], which promotes a more supportive environment for hematopoietic reconstitution [2,5]. This is in accordance with the data that surgical removal of the adipocyte-rich marrow induces hematopoietic infiltration and new osteoid and trabecular bone formation in rabbit tibias [6]. Considering that adipocytes and osteoblasts originate from the common precursor, mesenchymal stem cells (MSCs), within the bone marrow, where both display an inverse or reciprocal relationship [7], and that ATO could regulate the adipogenic and osteogenic differentiation of MSCs by significantly inhibiting adipogenic differentiation and enhancing MSCs osteogenic differentiation [8], ATO might be used to improve hematopoiesis in SAA patients.

Recently, we administered arsenic trioxide (ATO) plus cyclosporine in patients with SAA. The overall complete response rate and overall response rate at 17 weeks after the initiation of treatment were 80% (8/10) and 100% (10/10), respectively [9]. This observation prompted us to investigate whether ATO has activity in patients with SAA who have persistent, severe cytopenia after one or more cycles of immunosuppressive therapy. In this study, all patients achieved clinically significant responses to ATO. Therefore ATO could represent a reasonable salvage treatment in those patients with refractory SAA. The current study is being expanded to gain more data on this novel approach.

## Competing interests

The authors declare that they have no competing interests.

## Author contributions

Study concept and design: BF and NL; Acquisition of data: NL, YS, JZ and BF. Analysis and interpretation of data: BF, NL and YS. Drafting of the manuscript: NL, YS, JZ and BF. All authors have read and approved the final manuscript.
